# Additive effect between homocysteine and low-density-lipoprotein cholesterol upon incidence of novel carotid plaque formation: data from a Chinese community-based cohort

**DOI:** 10.1186/s12872-023-03282-z

**Published:** 2023-06-30

**Authors:** Mohetaboer Momin, Fangfang Fan, Ying Yang, Jianping Li, Jia Jia, Yan Zhang

**Affiliations:** 1 Department of Cardiology, Peking University First Hospital, 8thXishiku Road, Beijing, 10003 China; 2 Institute of Cardiovascular Disease, Peking University First Hospital, Beijing, China; 3 Echocardiography Core Lab, Institute of Cardiovascular Disease at Peking University First Hospital, Beijing, China

**Keywords:** Homocysteine, Carotid plaque, LDL-C, Atherosclerosis

## Abstract

Homocysteine (HCY) has been associated with carotid plaque in cross-sectional studies, but the prospective relationship between HCY and incident carotid plaque has not been well established. The purpose of this study was to investigate the association between HCY and incidence of novel carotid plaque in a Chinese community-based population without pre-existing carotid atherosclerosis and to assess the additive effect of HCY and low-density lipoprotein cholesterol (LDL-C) on the incidence of novel plaque. Methods: At baseline, we measured HCY and other risk factors in subjects aged ≥ 40 years. All participants underwent carotid ultrasound examinations at baseline and after an average of 6.8 years of follow-up. Incidence of plaque was identified if plaque was absent at baseline, but plaque was detected at the end of follow-up. A total of 474 subjects were included in the analysis. Results: The incidence of novel carotid plaque was 24.47%. Multivariate regression analyses showed that HCY was independently associated with a 1.05-fold-higher likelihood for incident novel plaque (adjusted odds ratio [OR] = 1.05, 95% confidence interval [CI]: 1.01–1.09, *P* = 0.008). Using tertile 1 and tertile 2 for reference, the top HCY tertile (T3) showed a 2.28-fold-higher likelihood for incident plaque (adjusted OR = 2.28, 95%CI: 1.33–3.93, *P* = 0.002). The combination of HCY T3 and LDL-C ≥ 3.4 mmol/L had the highest risk for novel plaque formation (adjusted OR = 3.63, 95%CI: 1.67–7.85, *P* = 0.001) compared to those without either condition. In the LDL-C ≥ 3.4 mmol/L subgroup, HCY was significantly associated with incidence of plaque (adjusted OR = 1.16, 95%CI: 1.04–1.28, *P* = 0.005, P-interaction = 0.023). Conclusion: In the Chinese community-based population, HCY was independently associated with the incidence of novel carotid plaque. There were additive effect between HCY and LDL-C on the incidence of plaque, the highest risk was observed in individuals with both high HCY levels and LDL-C ≥ 3.4 mmol/L. Our findings suggest that HCY may be a potential target for preventing the incidence of carotid plaque, particularly in individuals with elevated LDL-C levels.

## Background and aims

Atherosclerotic cardiovascular disease (ASCVD) is the leading cause of mortality globally. Novel carotid plaque formation is considered a marker of future carotid atherosclerotic disease and a well-accepted surrogate endpoint for ischemic stroke [1].

While traditional risk factors play a role in predicting atherosclerotic carotid disease [2–7], some well-established biomarkers, such as inflammatory, metabolic, endothelial, hematologic, lipids, and thrombosis-related biomarkers [8, 9], may also help in predicting the risk of atherosclerotic carotid disease. Low-density lipoprotein cholesterol (LDL-C) is proven to elevate ASCVD risk. However, some patients remain at significantly high residual risk despite intensive statin therapy [10].

Homocysteine (HCY) is well-documented as a biomarker for ASCVD [11]. Worth mentioning is that hyperhomocysteinemia (HHCY) is accounted for an increased risk of ischemic stroke in hypertension patients in China [12]. Studies have shown that HHCY was associated with carotid plaque presence, burden, instability, morphology, and total carotid plaque area (TPA) measured by Doppler ultrasound examination [13–15], as well as restenosis after carotid endarterectomy [7]. However, most of the prior studies regarding the association between HCY and carotid atherosclerosis were cross-sectional studies. Prospective data on HCY predicting plaque development in general populations were very scarce. and the cellular and molecular mechanisms linking HCY and atherosclerosis are not fully understood, which include oxidative stress, inflammation, DNA hypomethylation, endoplasmic reticulum stress, and mitochondrial dysfunction [16].

Previous studies also established a possible link between HHCY, dyslipidemia and atherosclerosis. An inverse association between HCY and high-density lipoprotein cholesterol (HDL-C) has been described in human and animal models of HHCY [17, 18]. Few studies showed that HHCY could accelerate atherosclerosis when associated with elevated plasma lipids [19]. However, the mechanisms linking HCY and lipid metabolism were not thoroughly investigated.

This study aims to investigate whether HCY is associated with future novel carotid plaque formation in Chinese community subjects without pre-existing carotid atherosclerosis. The study also aims to assess the additive effect between HCY and LDL-C on the incidence of novel carotid plaque. These findings may help in identifying individuals at higher risk of developing atherosclerotic carotid disease and inform the development of targeted prevention and treatment strategies.

## Methods

### Subjects

We enrolled subjects from a cohort in the Gucheng and Pingguoyuan communities of Shijingshan District in Beijing, China. At baseline (from December 2011 to April 2012), we measured HCY and other risk factors in subjects aged ≥ 40 years. All participants underwent carotid ultrasound examinations at baseline and after an average of 6.8 years of follow-up (from May 2018 to July 2018). The methods and preliminary results of this survey have been reported elsewhere [20, 21]. In brief, 708 participants with available data and quantitative carotid artery measurements for both baseline and follow-up were enrolled. Subjects at baseline with carotid plaques and CVD (self-reported coronary heart disease, stroke, or previous transient ischemic attack) were excluded. Finally, a total of 474 subjects were ultimately included in our analysis. This study was approved by Peking University First Hospital ethics committee. The procedures followed institutional guidelines and the Declaration of Helsinki principles, and each participant provided written informed consent.

### Measurements and definitions of the baseline data

We used a standardized questionnaire to interview participants to collect information, including their sociodemographic status, medical history, past and current medication use, and personal habits such as cigarette and alcohol consumption. We obtained a venous blood sample from every participant’s forearm after at least 12 h of overnight fast. These samples were used to measure the total cholesterol (TC), high-density lipoprotein cholesterol (HDL-C), low-density lipoprotein cholesterol (LDL-C), triglyceride (TG), fasting blood glucose (FBG), and the serum creatine (SCR) level. A Cobas 8000 modular analyzer (Roche Diagnostics, Indianapolis, IN, USA) was used for all laboratory variable measurements at baseline in the Peoples Liberation Army General Hospital laboratory. The estimated glomerular filtration rate (eGFR) was calculated using the CKD-EPI equation. The peripheral blood pressure was measured using the standard method, and we used the average of three measurements. Hypertension was defined as self-reported hypertension history, or systolic blood pressure (SBP) ≥ 140 mmHg or diastolic blood pressure (DBP) ≥ 90 mmHg or taking anti-hypertensive medicine. Diabetes mellitus was defined as self-reported diabetes history, or FBG level ≥ 7.0 mmol/L, or OGTT value ≥ 11.1 mmol/L, or taking anti-diabetic medicine. Dyslipidemia was defined as any self-reported history of hyperlipidemia, or TG level ≥ 1.70 mmol/L, or TC level ≥ 5.18 mmol/L, or LDL-C level ≥ 3.4 mmol/L, or HDL-C level < 1.04 mmol/L, or taking lipid-lowering medicine, according to the China Adult Dyslipidemia Prevention Guide (2007 Edition) criteria [22]. Current alcohol drinking was defined as drinking at least once per week for at least half a year, and current smoking was defined as smoking at least one cigarette per day for at least half a year. body mass index (BMI) was calculated using weight (kg) divided by height (m) squared. Current medication information for vitamin B supplements, antihypertension drug, anti-diabetic drug and lipid-lowering drug use were collected. The methods and relevant results of this data have been reported elsewhere [23, 24].

### Carotid ultrasonography measurement and definition for carotid plaque

All participants underwent carotid ultrasound examinations at baseline and after 6.8 years of follow-up. We used GE Vivid ultrasound system (GE Medical Systems, Milwaukee, WI, USA) equipped with an 8-MHz linear array vascular probe. Carotid ultrasounds were performed by the standard protocols according to Mannheim carotid intima-media thickness (CIMT) and Plaque Consensus [25]. The subjects were supine, with their head turned slightly away from the probe. We scanned the far wall and the near wall of the right and left common carotid artery, the bifurcation (bulb) and the internal carotid artery for the presence of plaques. A carotid plaque was defined using the Mannheim standard, as focal structures encroaching into the arterial lumen at least 0.5 mm or 50% of the surrounding IMT value or demonstrating a thickness > 1.5 mm as measured from the intima-lumen interface to the media-adventitia interface [25]. Certified sonographers performed carotid ultrasonography and judged the presence of a plaque. Incidence of plaque was identified if plaque was absent at baseline, but plaque was detected at the end of the study follow-up. The methods and relevant results of this data have been reported elsewhere [21].

### Statistical analysis

Categorical variables were presented by numbers and percentages. Continuous variables were described by using means plus standard deviations and medians for normally distributed data and non-normally distributed data, respectively. We conducted univariate comparisons between groups by using ANOVA for continuous variables and the χ2 test for categorical variables. We performed both univariate and multivariate logistic regression analyses to determine the relationships between HCY categories and the incidence of novel carotid plaque. We adjusted confounding variables in regression analysis, including age, gender, smoking, alcohol drinking, BMI, eGFR, TC, LDL-C, HDL-C, hypertension, diabetes, vitamin B supplements, antihypertension drug use, anti-diabetic drug use and lipid-lowering drug use (definitions mentioned above). Generalized additive model (GAM) with a spline smoothing curve was applied to inspect the relationship between the incidence of plaque and HCY, and a piecewise linear regression analysis was conducted to fit the smoothing curve. Stratification analysis and interaction terms were used to investigate whether the association between HCY and incident plaque differed according to LDL-C. Analyses were performed using Empower® (www.empowerstats.com, X&Y solutions, Inc., Boston, MA, USA) and R (http://www.R-project.org). *P* < 0.05 was considered statistically significant.

## Results

### Baseline characteristics of the study participants

In this study, 474 Chinese participants without carotid plaque at baseline were included. As Table 1 showed, the mean age of the participants was 51.36 ± 4.81 years, and 28.69% were male. The median level of HCY was 11.22 μmol/L. After an average follow-up period of 6.8 years, 116 of the participants developed carotid plaques, resulting in an incidence of 24.47%. The baseline characteristics of the novel plaque group were compared with those of the no plaque group, and significant differences were observed. The novel plaque group was found to be older, more likely to be male, and had lower eGFR and HDL-C levels. The prevalence rates of smoking, alcohol drinking, diabetes, and dyslipidemia were also higher in the novel plaque group. Importantly, the novel plaque group had a higher level of HCY (12.42 μmol/L vs. 10.94 μmol/L, *P* < 0.001) compared to the no plaque group.

**Table 1:** A comparison of the baseline characteristics of participants between Novel plaque group and No plaque group

	No plaque (*N* = 358)	Novel plaque (*N* = 116)	*P* value
Age(year-old), mean ± SD	50.86 ± 4.72	52.92 ± 4.75	< 0.001
Sex, N (%)			0.011
male	922 (25.70%)	44 (37.93%)	
famale	266 (74.30%)	72 (62.07%)	
eGFR(ml/min), mean ± SD	100.57 ± 9.12	97.80 ± 11.30	0.008
BMI (kg/m^2^), mean ± SD	25.78 ± 3.45	25.96 ± 3.08	0.616
TC (mmol/L), mean ± SD	5.27 ± 0.86	5.32 ± 1.05	0.655
LDL-C (mmol/L), mean ± SD	3.19 ± 0.69	3.29 ± 0.92	0.261
HDL-C (mmol/L), mean ± SD	1.50 ± 0.40	1.41 ± 0.38	0.029
TG (mmol/L), median(Q1-Q3)	1.17 (0.85–1.75)	1.42 (1.01–1.95)	0.264
SBP (mmHg), mean ± SD	127.92 ± 13.80	128.85 ± 14.86	0.594
DBP (mmHg), mean ± SD	75.42 ± 9.53	75.31 ± 8.99	0.915
FBG(mmol/L), mean ± SD	5.84 ± 1.33	5.96 ± 1.26	0.388
HCY(μmol/L), median(Q1-Q3)	10.94 (9.25–13.01)	12.42 (9.71–15.42)	< 0.001
Smoking, N (%)	48 (13.41%)	30 (25.86%)	0.002
Drinking, N (%)	76 (21.23%)	35 (30.17%)	0.048
Hypertension, N (%)	99 (27.65%)	35 (30.17%)	0.601
Diabetes, N (%)	41 (11.45%)	22 (18.97%)	0.038
Dyslipidemia, N (%)	232 (64.80%)	90 (77.59%)	0.010
Anti-hypertension drugs, N (%)	54 (15.25%)	18 (15.52%)	0.946
Anti-diabetic drugs, N (%)	20 (5.59%)	8 (6.96%)	0.588
Lipid-lowering drugs, N (%)	21 (5.95%)	10 (8.62%)	0.315
Vitamin B supplement, N (%)	23 (6.42%)	11 (9.48%)	0.267

### The smooth curve of the relationship between HCY and carotid plaque formation risk

**Fig. 1:**
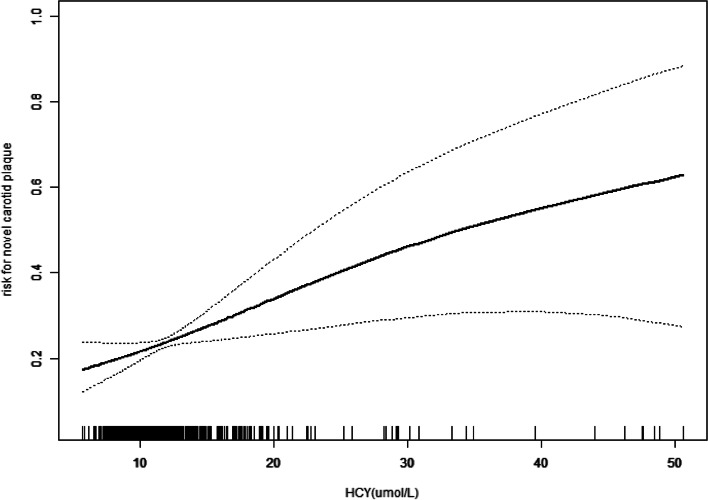
The smooth curve of the relationship between HCY and risk for novel carotid plaque formation. Adjusted for: age, gender, smoking, alcohol drinking, BMI, eGFR, TG, LDL-C, HDL-C, hypertension, diabetes, vitamin B supplement, antihypertension drug use, anti-diabetic drug use and lipid-lowering drug use

Figure 1 was the result of a generalized additive model (GAM) with a spline smoothing curve, which was applied to inspect the relationship between the incidence of plaque and HCY. The horizontal axis means the values of HCY, and the vertical axis means the predicted risk for novel carotid plaque. A higher HCY corresponded to a higher risk for novel carotid plaque formation after adjusting for age, gender, smoking, alcohol drinking, BMI, eGFR, TG, LDL-C, HDL-C, hypertension, diabetes, vitamin B supplement, antihypertension drug use, anti-diabetic drug use and lipid-lowering drug use. The dotted lines are the 95% confidence interval of the risk values.

### Univariate and multivariate regression analyses for HCY and incidence of novel carotid plaque

Univariate and multivariate regression analyses were conducted to investigate the potential independent association between baseline HCY and the incidence of novel carotid plaque. The results, which are presented in Table 2 using HCY levels and tertiles, were adjusted for several potential confounding factors, including age, gender, smoking, alcohol drinking, BMI, eGFR, TG, LDL-C, HDL-C, hypertension, diabetes, vitamin B supplement, antihypertension drug use, anti-diabetic drug use and lipid-lowering drug use. After controlling for these covariates, the odds ratios (ORs) for the incidence of novel carotid plaque increased with increasing HCY levels. Higher HCY was independently associated with a 1.05-fold increase in the likelihood of incident novel plaque (adjusted OR = 1.05, 95% confidence interval [CI]: 1.01–1.09, *P* = 0.008). When individuals in the top tertile of HCY (T3) were compared to those in the reference tertiles 1 and 2 (T1 + T2), they showed a 2.28-fold higher likelihood of developing novel carotid plaque (adjusted OR = 2.28, 95%CI: 1.33–3.93, *P* = 0.002).

**Table 2:** Univariate and multivariate regression analysis of the relationship between HCY category and incidence of novel carotid plaque

	Incidence, n(%)	Crude analysis		Multivariate analysis	
		OR (95%CI)	P	OR (95%CI)	P
HCY(μmol/L)	116 (24.5)	1.06 (1.03–1.09)	< 0.001	1.05 (1.01–1.09)	0.008
HCY Tertile					
T1(5.72–9.91)	32 (20.3)	1	-	1	-
T2(9.93–12.54)	28 (17.8)	0.85 (0.49–1.50)	0.585	0.81 (0.43–1.51)	0.506
T3(12.56–50.64)	56 (35.2)	2.14 (1.29–3.55)	0.003	2.00 (1.03–3.89)	0.040
T1-T2(5.72–12.54)	60 (19.1)	1	-	1	-
T3(12.56–50.64)	56 (35.2)	2.31 (1.50–3.55)	< 0.001	2.28 (1.33 -3.93)	0.002

Adjusted for: age, gender, smoking, alcohol drinking, BMI, eGFR, TG, LDL-C, HDL-C, hypertension, diabetes, vitamin B supplement, antihypertension drug use, anti-diabetic drug use and lipid-lowering drug use

### Additive effect and interaction analysis between HCY and LDL-C on the incidence of novel carotid plaque

Table 3 presents the results of the analysis that examined the joint effect of HCY and LDL-C on the occurrence of novel plaque. The analysis was adjusted for potential confounding variables, including age, gender, smoking, alcohol drinking, BMI, eGFR, TG, LDL-C, HDL-C, hypertension, diabetes, vitamin B supplement, antihypertension drug use, anti-diabetic drug use and lipid-lowering drug use. The study created four new categories based on the combination of HCY and LDL-C levels, including Group 1 (HCY < T3 and LDL-C < 3.4 mmol/L), Group 2 (HCY < T3 and LDL-C ≥ 3.4 mmol/L), Group 3 (HCY ≥ T3 and LDL-C < 3.4 mmol/L), and Group 4 (HCY ≥ T3 and LDL-C ≥ 3.4 mmol/L). The results indicated that participants with both elevated HCY and LDL-C (Group 4) had a 3.63-fold higher risk of developing novel plaque compared to those in Group 1 (adjusted odds ratio (OR) = 3.63, 95% confidence interval (CI): 1.67–7.85, *P* = 0.001). Furthermore, individuals with elevated HCY and normal LDL-C (Group 3) were 2.15 times more likely to develop novel plaque compared to those without either condition (adjusted OR = 2.15, 95%CI: 1.10–4.18, *P* = 0.025). In contrast, those with high LDL-C but normal HCY (Group 2) did not significantly affect the likelihood of developing novel plaque (*P* < 0.05) compared to Group 1.

**Table 3:** Additive Effect analysis between HCY and LDL-C on incidence of novel carotid plaque

		Crude analysis		Multivariate analysis	
		OR (95%CI)	P	OR (95%CI)	P
Group 1	HCY < T3 and LDL-C < 3.4 mmol/L	1.0	-	1.0	-
Group 2	HCY < T3 and LDL-C ≥ 3.4 mmol/L	1.52 (0.86–2.70)	0.152	1.51 (0.82–2.77)	0.186
Group 3	HCY ≥ T3 and LDL-C< 3.4 mmol/L	2.53 (1.46–4.38)	0.001	2.15 (1.10–4.18)	0.025
Group 4	HCY ≥ T3 and LDL-C ≥ 3.4 mmol/L	3.42 (1.73–6.75)	< 0.001	3.63 (1.67–7.85)	0.001

Adjusted for: age, gender, smoking, alcohol drinking, BMI, eGFR, TG, HDL-C, hypertension, diabetes, vitamin B supplement, antihypertension drug use, anti-diabetic drug use and lipid-lowering drug use

Table 4 presents the results of the analysis that explored the additive effect and interaction between HCY and LDL-C on the incidence of plaque. Specifically, the analysis examined the association between HCY and plaque incidence in the subgroup of participants with LDL-C ≥ 3.4 mmol/L. The results showed that HCY was significantly associated with the incidence of plaque in this subgroup (adjusted OR = 1.16, 95%CI: 1.04–1.28, *P* = 0.005), and the P-value for interaction was 0.023.

**Table 4:** Interaction analysis between HCY and LDL-C on the incidence of novel carotid plaque

HCY	LDL < 3.4 mmol/L		LDL ≥ 3.4 mmol/L		
	OR (95%CI)	P	OR (95%CI)	P	P interaction
Crude analysis	1.05 (1.01- 1.08)	0.009	1.12 (1.03, 1.21)	0.006	0.107
Multivariate analysis	1.03 (0.99- 1.07)	0.1632	1.16 (1.04, 1.28)	0.005	0.023

Adjusted for: age, gender, smoking, alcohol drinking, BMI, eGFR, TG, HDL-C, hypertension, diabetes, vitamin B supplement, antihypertension drug use, anti-diabetic drug use and lipid-lowering drug use

## Discussion

The current study aimed to investigate the potential association between HCY levels and the incidence of novel plaque formation in a previously carotid plaque-free population. The findings of this study revealed that HCY was independently associated with the incidence of novel plaque formation, and these associations differed according to LDL-C levels. Specifically, the correlation between HCY and plaque formation was more significant in individuals with high LDL-C and HCY levels. To the best of our knowledge, this is the first prospective study that has examined the relationship between HCY and carotid plaque formation in the general population.

Firstly, the results of the current study demonstrated a linear relationship between HCY levels and the incidence of carotid plaque formation. However, statistical significance was observed only in the top tertile of HCY (≥ 12.56umol/L), which may be due to the small sample size. Thus, further research based on a larger sample size is warranted to confirm the results of this study.

The relationship between homocysteine (HCY) and atherosclerosis is still a subject of debate in the medical field. Several cross-sectional studies have reported a significant correlation between carotid atherosclerosis and elevated HCY levels, while others have yielded conflicting results. Results from The Cyprus Study found that elevated HCY levels were associated with total plaque thickness in the Middle East population [26]. The CUDAS study showed that HCY was an independent risk factor for increased carotid plaque thickness in a general population from Western Australia [27]. Ryuichi et al. confirmed that the association between HCY and carotid plaque was present in elderly Japanese as well [28]. A study from Southern China revealed that HHCY aggravated instability of carotid plaque and carotid artery stenosis through increasing inflammation [29]. The Northern Manhattan Study also reported an association between elevated HCY and carotid plaque morphology and increased TPA [13]. Moreover, the findings from the Chinese population suggested that HCY was linked to advanced carotid plaque [14]. Results in Japanese showed that higher HCY levels correlated with increased severity of carotid atherosclerotic plaques and prevalence of lacunar infarction [30]. Similar results were found in chronic hemodialysis patients [31] and hypertensive patients [32]. In contrast, numerous cross-sectional studies didn’t find a significant association between HCY and carotid plaque in healthy French volunteers [33], non-insulin-dependent diabetes mellitus subjects [34], Chinese population [35, 36], Spain population [15], and patients with chronic renal failure [37].

Numerous prospective studies have explored the risk factors for carotid plaque progression [2, 4–6]. However, the prognostic role of HCY in carotid plaque remains unclear. Previous cohort studies on HCY and plaque progression in systemic lupus erythematosus patients have demonstrated a positive correlation between high HCY concentrations and atherosclerosis progression [38–40]. However, to our knowledge, this is the first cohort study investigating the association between HCY and plaque progression in the general population, thereby providing valuable evidence to resolve this knowledge gap.

Furthermore, our study found additive effect between HCY and LDL-C on the incidence of novel carotid plaque formation. The risk for carotid plaque progression was more significant in those combined HHCY and high LDL-C than either alone. We also analyzed the interaction between HCY and different levels of LDL-C. The additive effect was significant when LDL ≥ 3.4 mmol/L, whereas LDL2.6 ≥ mmol/L was not. Although the cut-off value of LDL-C is different according to individual cardiovascular risk, the results suggested that the combined effect of LDL-C and HCY was significant at a higher LDL-C level. These findings were consistent with previous studies. A study including 12,683 US subjects showed that individuals with elevated HCY and hypercholesterolemia have a higher likelihood to suffer a stroke than those without either condition [41]. W Herrmann et al. revealed that HCY improved the predictive value of LDL-C for coronary disease [19]. Data from a multicenter European case–control study showed that HCY increased the CVD risk associated with hyperlipidemia [42]. A case–control study of Korean men showed that metabolic syndrome with HHCY had a more excellent predicting value for CVD than metabolic syndrome alone [43]. Regarding experimental studies, several findings implied that HHCY enhanced and accelerated atherosclerosis induced by hyperlipidemia [44–46].

The present study has some limitations that need to be acknowledged. Firstly, only a single baseline measurement of plasma HCY was used to predict future cardiovascular events. Subsequent changes in plasma HCY levels were not considered, which may have influenced the results. Secondly, the study sample was relatively small and was recruited from an urban community in northern China, which may limit the generalizability of the findings to other populations. Moreover, other factors that could contribute to plaque progression, such as hsCRP, were not taken into account. Additionally, other indexes of carotid atherosclerosis, including TPA and plaque number, were not analyzed in this study.

Despite these limitations, our study provides significant implications for the prevention and assessment of CVD. Specifically, our findings suggest that testing HCY levels, especially in individuals with high LDL-C, is crucial for a comprehensive evaluation of CVD risk. By identifying individuals with elevated HCY levels, appropriate interventions, such as lifestyle modifications and pharmacological therapies, can be initiated to reduce the risk of future cardiovascular events. These findings have important implications for healthcare providers and policymakers, as they highlight the need for targeted screening and prevention strategies to reduce the risk of cardiovascular disease in high-risk populations. Further studies with larger sample sizes and more comprehensive assessments of atherosclerosis are needed to confirm our findings and guide clinical practice.

## Conclusion

In conclusion, this 6.8-year follow-up study showed that HCY was independently associated with novel carotid plaque formation in persons with no plaque at baseline. Combining LDL-C with HHCY increased the predictive strength for novel carotid plaque. The findings of this study suggest that it is crucial to comprehensively screen for HCY in populations with high LDL-C to prevent atherosclerosis. Therefore, interventions targeting HCY levels may be necessary in individuals at risk of developing atherosclerosis.

## Data Availability

The datasets used or analyzed during the current study are available from the corresponding author upon reasonable request.
